# Periscope

**Published:** 1888-12

**Authors:** 


### PERISCOPE.

### MEDICINE.

**Sulphonal.**—Dr. Garnier has given this new hypnotic an extended trial in the treatment of the insane, and considers it superior to paraldehyde, urethane, methylal, or chloral. He gives it in doses of 2—5 grammes, suspended in some menstruum, since it is insoluble in cold water, and re-precipitated on cooling from its solution in boiling water.

M. Mathes, a pupil of Professor Ziemssen, has lately published a thesis on Sulphonal. He gave it indiscriminately to patients suffering from all kinds of diseases, without observing any untoward results. Patients with cardiac affections were able to take as large a quantity as those suffering from pulmonary phthisis. He tabulated his results thus: Sulphonal has a complete soporific effect in 72 per cent., incomplete in 9 per cent., and none at all in 18 per cent. In 19 per cent. it gives rise to accessory symptoms, such as noises in the ears, dizziness, slight headache, general fatigue, and, exceptionally vomiting. No disturbance of the respiratory, circulatory or digestive systems was observed. M. Mathes concludes—

1. That sulphonal is an useful hypnotic, though not always absolutely certain.

2. It has no taste nor odour, and does not interfere with the functions of any of the chief organs.

3. It only produces bad effects in a small number of cases, and these, when present, are not serious.

4. The dose varies according to the individual susceptibility. The average dose is about a gramme; it may be increased if necessary. If it causes any disagreeable symptoms, the dose is to be reduced.

5. On account of the slowness of its action, it must be given at least an hour before the customary bedtime.

6. When insomnia results from cough, or from any but neuralgic pain, sulphonal is contra-indicated. In many true neuralgias it is most valuable.

The above statements have been confirmed by many other German observers.—*Le Progrès médical*, October 13th and 20th, 1888.

**Action of Hydrofluoric Acid on the Bacillus of Tubercle.**—M. Jaccoud read, at the Academy of Medicine, a paper on this subject, in which he stated that, as the result of numerous experiments on animals, he had come to the conclusion that hydrofluoric acid in solution in water, up to the strength of equal parts of each, does not suppress or lessen in any degree the virulence of sputum containing tubercle-bacilli, and that it is powerless to modify the vitality of the bacillus. M. Hérard disagreed with these statements, and thought that M. Jaccoud's error lay in assuming that pure hydrofluoric acid was too powerful in its action to be used in practice; he had found, on the contrary, that air charged with the vapour of pure hydrofluoric acid can be breathed for periods of one hour with impunity, in the minimum dose of one litre per minute. He cited the experiments of Trudeau, of New York, as absolutely proving the destructive action of hydrofluoric acid on the bacillus. Tube-cultures of tubercle-bacilli remained sterile, and did not give rise to tuberculosis in animals when inoculated, after they had been exposed, in one series of experiments, to solutions of from 1—100 to 1—400 of hydrofluoric acid in water, and, in another series, to air that had been previously passed through a 1—3 solution of the acid. Further, long-continued inhalations of hydrofluoric acid had given excellent results in rabbits. M. Hérard was convinced of the value of these inhalations, and gave several examples of the good results obtained in his own practice. The remedy probably produces its beneficial action by increasing the appetite, by modifying the bronchial secretions, and so preventing the growth and multiplication of the bacilli in these secretions.

MM. Grancher and Chautard, in studying the same question, concluded that in experimental tuberculosis the action of the hydrofluoric acid vapour was *nil*. M. Jaccoud cited these observations in support of his view; but M. Hérard pointed out that, in such experiments, the intra-venous method of inoculation employed leads to generalised tuberculosis, and that by inhalations one could not expect to act on bacilli disseminated through all the organs of the body.

In connection with the many specific methods of treatment of pulmonary phthisis lately advocated, it is interesting to compare the results given in a paper by Dr. Dettweiler, the head of the Sanatorium of Falkenstein (quoted in *Gazette des hôpitaux*). At Falkenstein the essence of the treatment lies in constant exposure, so far as possible, to a pure, exhilarating atmosphere, combined with a liberal dietary, an amount of physical exercise suitable to each case, with rest and symptomatic treatment of pyrexia, &c. Of 1022 cases thus treated, 13.2 per cent. were completely cured, and 11 per cent. much benefited.—*Le Progrès médical*, November 3rd; *Gazette des hôpitaux*, November 8th, 1888.

**Sea-sickness.**—As the result of an elaborate series of experiments on animals, M. Pampoukis, of Athens, recommends the adoption of the following measures in order to avoid sea-sickness: Before going on board, surround the abdomen with a broad belt, in order to prevent displacements of the abdominal viscera from the movements of the vessel. Avoid during the voyage liquid foods or soups as far as possible; if necessary, take two or three small doses of brandy in the day. At the first feeling of vertigo, go and lie down in the cabin, and take cocaine, which, according to the author's experience, prevents sickness, but not vertigo. The best preventive measure of all would be to suspend the beds in the same fashion as the lamps on board ship.—*Le Progrès médical*, October 6th, 1888.

**A Case of Subacute General Anterior Spinal Paralysis.**—MM. Pitres and Vaillard report a typical case of the affection described by Duchenne under this title. The patient, a robust man of 43 years of age, took a violent chill, which was followed by shivering fits, a feeling of general malaise, colic, and tenesmus with slight diarrhœa. A month later, another attack of the same kind came on, with extreme feebleness; and six weeks after the onset of the illness, he came under observation, complaining of general weakness. The muscular feebleness was very marked. Two days after admission, paralysis, without affection of sensation, came on in the right shoulder, and shortly afterwards in the left. About a week later, the paralysis had extended to the arms, and to the muscles on the posterior aspect of the forearms. The patient now complained of great weakness in the legs; was barely able to stand, and could not walk at all. Seven weeks after admission, all motor power in the four limbs was completely lost. The muscles now began to waste, and to lose their excitability to the Faradic current in the order in which they had been involved in the paralysis. There was no affection of sensation, nor of the sphincters, nor of the organs of special sense, and no fever. A month later, there was œdema of hands and of the lower extremities, but no albuminuria. Slight signs of bulbar paralysis now showed themselves, with nocturnal delirium. Two months afterwards, the patient was in the same condition, except that he was beginning to gain power in the right arm: at this time he died of an attack of lobar pneumonia.

At the autopsy, the brain and spinal cord were normal; the only abnormality found, on microscopic examination of the spinal cord, was a slight degree of thickening of the neuroglia in the lateral columns and in the columns of Goll. The peripheral nerves, both in the large nerve-trunks and their finest ramifications, showed considerable morbid changes of the form described as "degenerative neuritis," differing in degree only, and not in the nature of the pathological alterations, in different regions. Briefly, the changes consisted in breaking up or segmentation of the myelin sheaths (early stage), followed by complete atrophy, leaving empty nerve-sheaths mixed with finely varicose fibrils. The proportion of healthy nerve-fibres mixed with the atrophied fibrils in the nerve-trunks was exceedingly small. These lesions affected without exception the nerve-trunks and their branches in the four limbs. The anterior roots of the spinal nerves were much less altered. Individual nerve-fibres were found to be affected in the same way as the foregoing; but there were few diseased nerve-fibres, and these occurred individually, scattered amongst the very much larger proportion of healthy fibres, whilst here the morbid process never destroyed whole bundles of nerve-tubes as it did in the case of the peripheral nerves.

The symptoms of this affection resemble those of Landry's paralysis; but differ in the slowness of onset and progress. Duchenne thought that the seat of the lesion would be found in the motor cells of the anterior cornua of the spinal cord. The case is interesting, since it shows that in this undoubted instance of the affection in question, the peripheral nerves were affected almost alone, the slight change found in the spinal cord being unimportant. It also shows that profound alterations in the mixed nerves may cause only general motor paralysis without any affection of sensation. The authors give a brief account of the few cases of this rare affection that have been published hitherto.—*Le Progrès médical*, September 1st, 1888. J. MICHELL CLARKE, M.B.

### LARYNGOLOGY.

**The Persistence of Phonetic Troubles after the Removal of Post-nasal Adenoid Growths.**—Cartaz recommends that such patients should be made to articulate slowly and in syllables, and that they should read as if they were scanning. Also that they should practise the tonic sol-fa, sustaining the notes for some time. If these means are insufficient, he has recourse to electricity applied to the soft palate.—*Archives de Laryngologie*, December, 1887.

**The Treatment of Suppuration of the Ear.**—Verdos has obtained the best results from the use of powdered boracic acid or borax, used after the method of Bezold, in cases where the discharge is abundant, of long standing, and where there is no deep-seated mischief. If there exists periostitis, ostitis, or vegetations, he alternately uses boracic acid and nitric acid, very carefully applied.

Iodoform is also useful, in powder form, but not if hyperæmia is present. Its value is best seen in scrofulous cases, chronic or acute, and especially if there is ulceration of the outer part of the tympanic membrane, and of the external anditory meatus.

Corrosive sublimate, in $\frac{1}{2}$ to 2 per cent. solution in glycerine, is a "precious" drug; but it must not be used in the hyperæmic stage.

Carbolic acid he considers to be of small value, but useful for syringing in 3 per cent. strength.

Sulpho-carbolate of zinc, in 1 to 2 per cent. solution, is only of value for syringing in cases of auricular furuncle.

If there be no perforation, remedies must be made to pass into the middle ear, and preferably in gaseous form—*e.g.* iodine, benzoate of soda, turpentine, tincture of benzoin, &c.—and are most valuable, as also aeration of the tympanum.—*Revue mensuelle de Laryngologie*, November, 1888.

[There is no doubt as to the value of the boracic acid treatment; but the powdered acid will dry on and irritate the meatus, unless this is carefully dried after syringing, and vaseline applied to all parts where the local action of the acid is not wanted. Iodoform is of very little value, salicylic acid and rectified spirit, both of great value, have unaccountably been omitted by the author.]

**A Case of Laryngeal Stenosis due to Compression by Goitre.**—Beverley reports the case of a woman, age 60 years, who was seized with a suffocative attack due to compression of the recurrents by a goitre, which was cured by removal of the thyroid.—*New York Med. Journ.*, January 28th, 1888. [This is an uncommon cause of suffocation, only discoverable by using the laryngyscope.]

**Atrophic Coryza (Essential Ozæna).**—Dr. E. J. Moure, after going very fully into the etiology and pathology of this condition, lays down the following rules for treatment:

A first irrigation of the nostrils, made with one or two litres of tepid sulphurous water, to which is added either chlorate of potash, bicarbonate of soda, or borax, in the proportion of a teaspoonful to the litre. This is followed by a second irrigation, consisting of half a litre of tepid water, with the addition of a tablespoonful of the following fluid:

| | |
|---|---|
| Carbolic Acid ... ... | 20 parts |
| Glycerine ... ... ... | 100 ,, |
| Alcohol at 90° ... ... | 50 ,, |
| Water ... ... ... ... | 350 ,, |

In order that the patient may not get accustomed to this solution, he substitutes chloral, resorcin, salicylic acid, &c., for the carbolic acid every month. After the douches, the patient ends the treatment by spraying the nostrils or insufflation of fine powder. For spraying he uses:

| | | |
|---|---|---|
| | Carbolic Acid ... ... | 2 parts |
| | Resorcin (Crystals) ... | 3 ,, |
| | Glycerine ... ... ... | 50 ,, |
| | Water ... ... ... ... | 300 ,, |
| Or, | Camphor ... ... ... | 8 ,, |
| | Tinct. Iodi ... ... ... | 10 ,, |
| | Tar ... ... ... ... | 12 ,, |
| | Alcohol at 90° ... ... | 100 ,, |
| | Water ... ... ... ... | 200 ,, |

To be warmed and used to moisten the nose for one or two minutes after spraying.

Nasal douches must be carefully directed towards the naso-pharynx, and not up towards the root of the nose. They should be used tepid, and in small stream.

Thymol, Moure does not like, as it is painful and not certain in its action.

Surgical treatment he entirely discards, and Gottstein's tampons he has not found valuable, and very inconvenient.—*Journal of Laryngology and Rhinology*, November, 1888.

BARCLAY J. BARON, M.B.

